# Preparation and Characteristics of Alginate Microparticles for Food, Pharmaceutical and Cosmetic Applications

**DOI:** 10.3390/polym14183834

**Published:** 2022-09-14

**Authors:** Anna Łętocha, Małgorzata Miastkowska, Elżbieta Sikora

**Affiliations:** Faculty of Chemical Engineering and Technology, Institute of Organic Chemistry and Technology, Cracow University of Technology, 31-155 Krakow, Poland

**Keywords:** alginate, microparticles, active substances, probiotics, encapsulation techniques

## Abstract

Alginates are the most widely used natural polymers in the pharmaceutical, food and cosmetic industries. Usually, they are applied as a thickening, gel-forming and stabilizing agent. Moreover, the alginate-based formulations such as matrices, membranes, nanospheres or microcapsules are often used as delivery systems. Alginate microparticles (AMP) are biocompatible, biodegradable and nontoxic carriers, applied to encapsulate hydrophilic active substances, including probiotics. Here, we report the methods most frequently used for AMP production and encapsulation of different actives. The technological parameters important in the process of AMP preparation, such as alginate concentration, the type and concentration of other reagents (cross-linking agents, oils, emulsifiers and pH regulators), agitation speed or cross-linking time, are reviewed. Furthermore, the advantages and disadvantages of alginate microparticles as delivery systems are discussed, and an overview of the active ingredients enclosed in the alginate carriers are presented.

## 1. Introduction

“Microparticle” is the term used for spherical particles with diameters in the micrometer range, typically from 1 to 1000 µm. Polymeric microparticles are usually formed by a polymer matrix in which a smaller amount of an active compound could be immobilized [1]. Generally, taking into account the method of microparticle preparation, their morphology, and the distribution of the encapsulated actives, microparticles can be divided into two categories: “microspheres” and “microcapsules” [1,2,3,4].

Microspheres usually are characterized as matrix systems in which the active substance is homogeneously dispersed. In contrast, microcapsules are heterogenous particles where a membrane shell surrounds the core (solid or liquid) and forms a reservoir with an encapsulated active compound [1,5,6]. In some cases, to overcome problems with the mechanical stability of such, the carriers and low-actives-loading, polymer-coated microspheres are obtained [7,8].

Compared with other effective carriers of the active compounds, such as nanoparticles, the advantage of the microcarriers is that they do not penetrate into the *interstitium*, and thus act locally [5,9]. The choice of microcapsules over nanocapsules in the case of cosmetic products can be an ideal solution for epidermal action, where the encapsulated ingredient is designed to act on the top of the *epidermis*. Moreover, nanocapsules are not suitable, due to their small size, to encapsulate active ingredients with larger than nano sizes, including bacteria.

The microparticles can be prepared from a large variety of starting materials, both natural and synthetic origin, and with different preparation techniques. Most drug delivery systems are prepared using natural polymers, such as polysaccharides (e.g., starch, dextran, alginate, hyaluronic acid and chitosan) or proteins (e.g., collagen, gelatin and albumin) [10,11]. Among synthetic polymers applied to obtain the carriers, there are poly(lactide-co-glycolide) (PLGA), (3-hydroxybutyrate-co-3-hydroxyvalerate) (PHBV), poly(sebacic anhydride) and poly(ε-caprolactone) [2]. Moreover, thermosensitive polymers, e.g., poly(N-isopropyacrylamide) (PNIPAAm) [12], and pH-sensitive polymers (Eudragit L100 and Eudragit S100) are used [13,14].

Among the natural raw materials used to obtain microparticles, alginates are the most popular and deserve special attention. In the food industry, alginate-based formulations are used as texture modifiers [15,16,17] or to improve the stability and long-term efficacy of active compounds [18,19,20]. In the case of the cosmetic and pharmaceutic industries, alginate systems are used to improve stability and to protect the encapsulated compounds against external conditions, e.g., UV light, temperature [21,22,23], or gastric environments in the case of oral application [24,25,26,27]. The alginate microparticles (AMP) are biocompatible, biodegradable and nontoxic delivery systems allowed to encapsulate different active substances, including probiotics [28,29,30].

The objective of this article was to provide a review of the techniques used for AMP production. The most popular alginate encapsulation methods, such as the spray-drying technique, extrusion and emulsification are described. The technological parameters important in the microencapsulation process and their impact on the quality of the alginate carriers are discussed. Particular attention was paid to the microencapsulation of alginates based on the emulsification technique, as the process allows to obtain small microparticles, containing both hydrophilic and hydrophobic ingredients with high efficiency. Moreover, the examples of the actives enclosed in the AMP, and their application in the food, pharmaceutical and cosmetic industries, are presented. Most scientific articles focus on applications of AMP in the food and pharmaceutical industries [28,31,32,33]. Recently, the environmental application of alginate carriers has also appeared more often [34]. However, the cosmetics industry has not been the subject of wide scientific interest so far.

## 2. Alginate Characteristics

**Figure 1:**
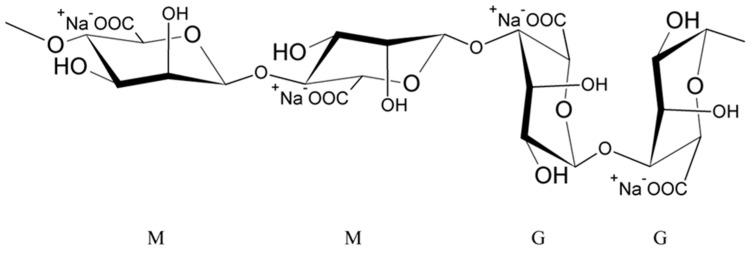
Structure of sodium alginate.

Alginates are natural polysaccharides, polyanionic polymers, obtained from marine algae [35,36], usually from three species of marine brown algae, Phaeophyceae: *Macrocystis pyrifera*, *Laminaria digitata* and *Laminaria saccharina*. The structure of alginates depends on the source of the sea algae, i.e., its species, geographic origin, or seasonal varieties [37]. Alginates are unbranched copolymers of D-Mannuronic acid (M block) and L-Guluronic acid (G block) linked by β (1–4) glycosidic bonds [37,38,39,40,41,42,43]. These blocks are arranged in an irregular block pattern with different GG, MM and MG block proportions (Figure 1) [30,35,37,38,42]. Blocks M and G are placed in the different locations along the chain (e.g., MMMM, GGGG, MMGG, GMGM) and in various amounts [38].

### Cross-Linking of Alginates

**Figure 2:**
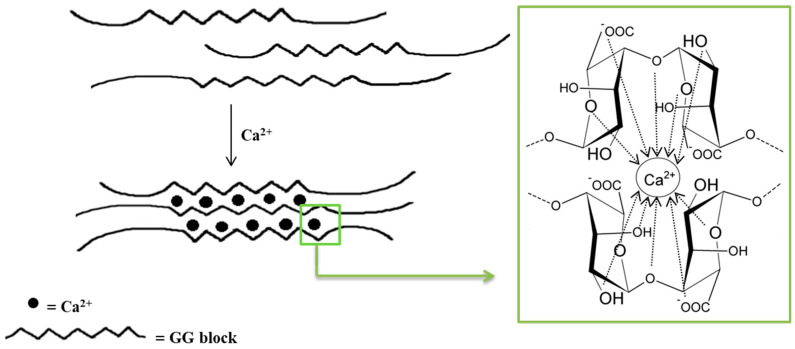
Scheme of an alginate gel formation by calcium cations (“egg–box” model).

Monovalent metal ions with alginates form soluble salts, while divalent and multivalent cations (except Mg^2+^) form gels or precipitate. The affinity of alginates to various cations and selective ion binding are the basis of the alginates’ ability to form hydrogels. Alginates containing a large number of guluronic acid blocks form gels with much greater strength compared with mannuronate-rich alginates because G blocks have a stronger affinity with divalent ions than M blocks [35]. The interaction of alginates with divalent cations, which are in particular calcium cations, leads to the formation of biodegradable gels [38]. Polymerization is based on the cross-linking of copolymers through ionic bonds between Ca^2+^ cations and alginate anions [38,44]. Each Na^+^ cation ionically binds to only one carboxyl group of the alginate chain, while the Ca^2+^ cation interacts with two carboxyl groups that come from different polymer chains. The exchange of Na^+^ ions into Ca^2+^ ions is relatively easy and takes place when an aqueous solution of sodium alginate is mixed with a solution containing calcium ions [41]. The mechanical properties of the formed gels are determined by both the concentration of divalent ions and the alginic acid salts in the reaction mixture [38,44]. The distinctive molecular structure resulting from these interactions is referred to as the “egg–box” model (Figure 2) [38,44,45,46]. It should be underlined that only the G blocks take part in the cross-linking process. The homopolymers of G blocks form ordered, three-dimensional regions wherein Ca^2+^ ions are embedded like eggs in a cardboard box [47].

The immediate cross-linking of alginates, due to the action of calcium ions, causes the formation of particles of different diameters and different porosity [48]. The gel strength increases with the increase in G blocks content. Moreover, the parameters of the cross-linking process are important. Temperatures ranging from 60 to 80 °C are needed to dissolve alginates in the water. Moreover, it is known that alginate gels are insoluble in acidic environments [49]. Additionally, the technique used to obtain alginate particles influences the capsule size, which can range in size from nanoparticles to macroparticles. Macroparticles are particles larger than 1000 microns in size and are easily visible [50,51]. They are applied especially in the field of dietary supplements [52] or drugs [53,54,55,56]. The size of microparticles ranges from 100 nanometres to 1000 μm [57,58,59], and particles with a diameter below 100 nanometres are classified as nanoparticles [60].

## 3. Alginate Encapsulation Techniques

Generally, microencapsulation techniques are classified into three groups [61,62,63]:

(a) Physical methods such as spray drying, extrusion, lyophilization, supercritical fluid precipitation and solvent evaporation;
(b) Physico-chemical methods including coacervation, liposomes and ionic gelation;
(c) Chemical methods such as interfacial polymerization and molecular inclusion complexation.

In the case of alginate capsules preparation, the most frequently used methods are the spray-drying technique and either extrusion or emulsification/gelation [61].

### 3.1. Spray-Drying Technique

**Figure 3:**
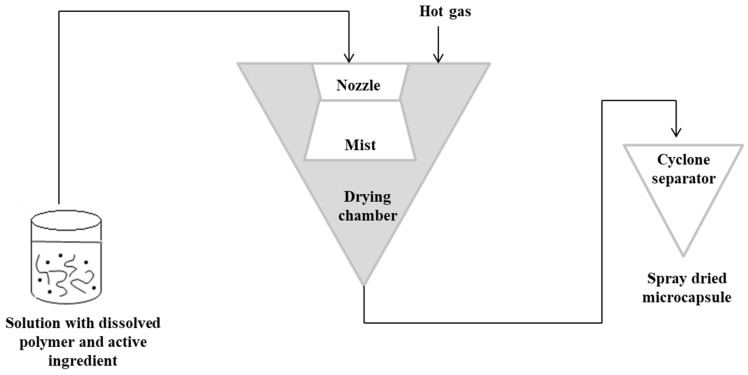
Scheme of the spray-drying encapsulation technique.

The spray-drying technique is the oldest technique used in alginate capsules. The concept of this method was first described in Samuel Percy’s patent in 1872 [64]. The process was introduced commercially in the 1920s, but spray drying was fully applied on a large scale in the early 1980s [65,66]. In this method, initially, a solution containing the active ingredient should be prepared with the dissolved polymer matrix. The solution is then pressurized and sprayed to form a “mist” in the drying chamber. Hot gas (air or nitrogen) is also blown into the drying chamber. The hot gas makes it possible to evaporate the solvent. The capsules are then transported to a cyclone separator for recovery [3]. In this technique, the control of the product’s feed, gas flow and temperature is important [67,68]. Figure 3 shows a schematic representation of the spray-drying procedure [3,63].

The spray-drying technique is applicable to encapsulate different kind of actives. Among substances encapsulated in alginate capsules, there were lipids [69] and hydrocarbons such as cellulase [70], carvacrol [71], or insulin [72]. Unfortunately, the main disadvantage of this method is the impossibility to encapsulate substances sensitive to elevated temperature and pressure.

### 3.2. Extrusion Technique

Another technique that has been used for a long time for producing microparticles is the extrusion technique [73]. It consists of the preparation of a hydrocolloid solution (an aqueous dispersion of a natural or synthetic polymer), e.g., sodium alginate. Then, the material for encapsulation is added to the solution, and the suspension is pressed into droplets into the gelling or hardener solution, e.g., calcium chloride [3,30,39,49,67,74,75,76,77]. Depending on the scale (laboratory or pilot scale), a syringe needle or an extruder is used for the process, respectively [39]. The size and shape of the obtained beads depend on factors such as the diameter of the needle and the distance of the needle from the hardener solution. This technique is the most popular in laboratory scale because of simplicity and low cost of production [78]. However, the main limitation in the case of its application in the large scale is the fact that microparticles are formed slowly [30].

### 3.3. Emulsification Technique

**Figure 4:**
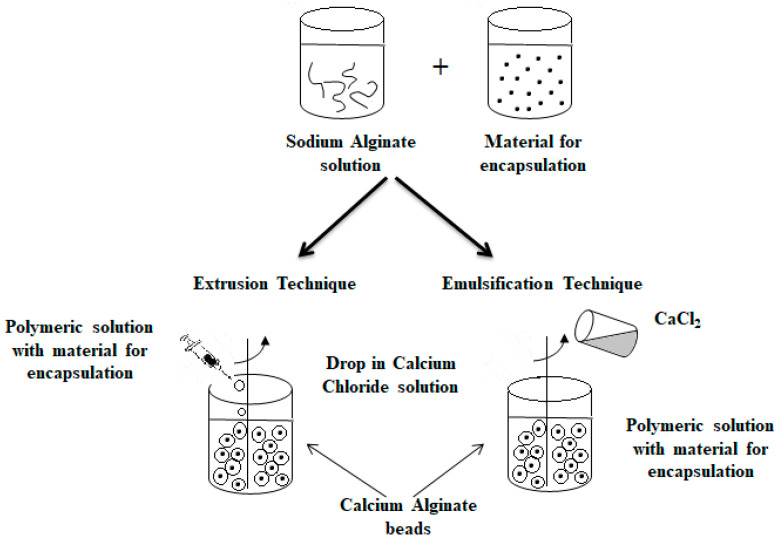
Scheme of the encapsulation process by extrusion (on the (**left**)) and by emulsification technique (on the (**right**)).

An alternative method commonly used to obtain alginate capsules is emulsification. The emulsification technique is more expensive than the extrusion method because a large amount of oil is needed to prepare the emulsion [79]. In this technique, the discontinuous phase (mixture of hydrocolloid and encapsulated material) is added to a large volume of continuous oil phase oil [3,30,39,46,49,67]. In the case of food applications, vegetable oils are used as a continuous phase, most often canola, sunflower or corn oils [78,80]. Additionally, to stabilize the droplets of the internal phase, an emulsifier should be added to the mixture formed. The obtained water-in-oil emulsion is continuously homogenized by stirring [3,30,39,49,67]. The speed of the emulsion mixing is a critical step because it affects both the shape and size of the capsules formed [81]. Very large capsules (around 1000 μm in diameter or larger) can result in poorly coated structures and a coarse texture [82], which in turn affects the dispersion quality of the capsules in the final product [79]. The final emulsion containing alginate droplets is broken by adding calcium chloride solution and centrifuged, thereby separating the oil and water phases to obtain microspheres [83,84]. Figure 4 shows a diagram of the encapsulation process using extrusion and emulsification techniques [46,67].

The main difference between the two techniques is the sequence of combining the alginate and calcium chloride solutions. The polymer solution containing the active ingredient is introduced into the calcium chloride solution in the case of the extrusion technique as opposed to the emulsification technique, where the CaCl_2_ solution is dripped into the polymer solution. Depending on the method used to obtain them, the calcium alginate beads are called balls (gelled droplets obtained by extrusion technique) or capsules, in the case of the products of the emulsification technique [85]. The main differences between these alginate beads are listed below [49,78,85,86]:

- The balls have a porous network, while the capsules have a liquid core (water or oil);
- The dimensions of the balls are much larger than in the case of the capsules;
- The balls are uniform in size and shape in contrast to differing in size in capsules.

The differences in the morphology (structure) of both the microparticle types are perfectly illustrated in images obtained by electron microscopy TEM and SEM [87].

To sum up, some advantages and disadvantages of the above-presented methods of AMP preparation are summarized in Table 1.

The data presented in Table 1 indicate that the currently used alginate encapsulation techniques are not universal. All methods have some pros and cons, but among the others, the emulsification is more universal. The method allows to obtain small microparticles, containing both hydrophilic and hydrophobic ingredients with high process efficiency. Moreover, the microencapsulation of alginates based on the emulsification technique could be modified by differing the polymer gelling process.

## 4. Microencapsulation of Alginates Based on Emulsification Technique

The emulsification process is based on the mechanism of external or internal gelling. Both water-in-oil (W/O) and oil-in-water (O/W) emulsions could be used to prepare microparticles. Depending on the gelling system used, the obtained microparticles can have different properties and thus different applications. The properties differentiating the external and internal gelling are: pore size, stiffness, permeability and strength of the matrix. Alginate microspheres prepared with external gelling have smaller pores and a denser surface structure than inside the capsule. In turn, microspheres obtained by internal gelling have a more homogeneous structure, which is associated with a more uniform distribution of cations in the particle [91,92,93,94].

### 4.1. Alginate Microspheres Formed in a W/O Emulsion—External Gelation

In the external gelation emulsification method, an alginate solution containing the encapsulated active substance is emulsified in the oil phase to form a W/O emulsion. Next, a cross-linking compound, e.g., calcium chloride solution, is gradually added to the emulsion [42,95,96,97]. The addition of CaCl_2_ solution causes the droplets of the alginate emulsion to gel and form microspheres [4,98]. The calcium chloride particles in the solution migrate according to the principle of diffusion to the phase interface of the droplets of the alginate emulsion (the dispersed phase), where they dissolve in the aqueous phase and cause gelation to form the microspheres [98]. This step is followed with filtration and rinsing to remove residual oil. In this method, the obtained microspheres consist of a soft core and a rigid outer matrix [29,99].

### 4.2. Alginate Microspheres Formed in a W/O Emulsion—Internal Gelation

In an internal gelation process, insoluble calcium crystals (e.g., calcium carbonate) are dispersed in an aqueous polysaccharide solution and serve as an internal calcium source. This mixture is emulsified in an oil phase containing a surfactant. Upon pH reduction, from 7.5 to 6.5, calcium (Ca^2+^) is released from the calcium complex causing gelation to form calcium alginate. To lower the pH, usually an oil-soluble organic acid such as acetic acid is gently added to the oil, causing immediate diffusion into the water phase. In this way, the immediate lowering of the pH of the dispersed droplet, solubilizing the crystalline calcium, causes rapid gelling [4,29,98,100].

Considering the gelation process, two successive reactions follow after diffusion of the oil-soluble acid through the oil–water interface. Protons that diffuse into the aqueous gel phase encounter evenly spaced calcium microcrystals, usually CaCO_3_, which causes the immediate release of calcium ions (1) and in situ gelling of the alginate, creating a homogenous gel network (2). Below is the course of the reactions taking place [29,100]:

2H^+^ + CaCO_3_ → Ca^2+^ + H_2_O + CO_2_ (1)

Ca^2+^ + Na^+^G → Ca^2+^G + Na^+^ (2)

Internal gelation produces symmetrical microspheres with large pores and a low matrix density compared with external gelation [29,101].

**Figure 5:**
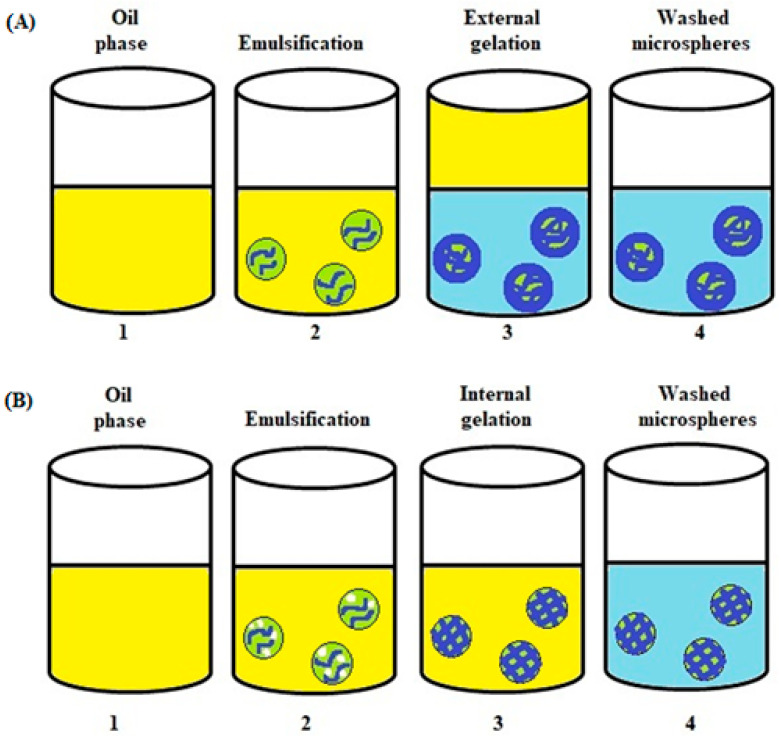
Schematic representation of microsphere formation with emulsification coupled with external (**A**) and internal gelation (**B**). (**A**) 1. Continuous oil phase. 2. Emulsifying an alginate solution in the oil phase (creating a W/O emulsion). 3. Addition of CaCl_2_ solution to the oil phase (external gelation of the alginate microspheres). 4. Washed alginate microspheres. (**B**) 1. Continuous oil phase. 2. Emulsifying an alginate solution (containing an insoluble calcium source) in the oil phase (creating a W/O emulsion 3. Solubilization of the calcium source by adding acid and lowering the pH inside the beads (internal gelation of the alginate microspheres). 4. Washed alginate microspheres.

A schematic representation of microsphere formation with internal and external gelation is shown in Figure 5 [4,29,98].

### 4.3. Gelation at the Interfacial Area of Emulsion (Complexation)

**Figure 6:**
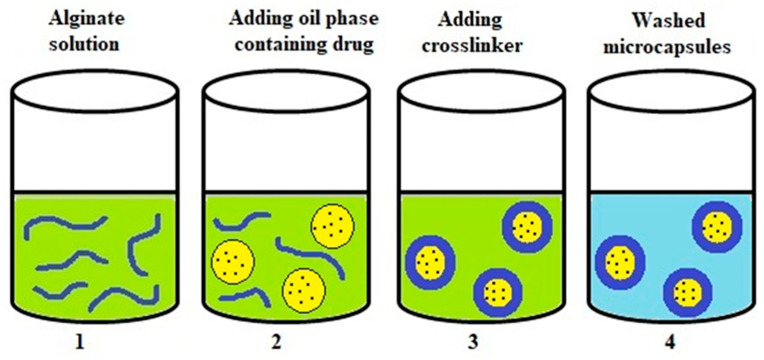
Schematic representation of microcapsule formation. 1. Alginate solution. 2. The oil phase, containing the encapsulating ingredients, is emulsified in the alginate solution. 3. The addition of CaCl_2_ solution and formation of alginate microcapsules at the interface of the oil droplets. 4. The solvent is removed, and the microcapsules are isolated and washed.

One method of forming an emulsion is to deposit the polymer at the interface of the emulsion droplets and then remove the solvent used [102]. The polymer coating that deposits the polymer at the interface is stabilized with covalent or physical intermolecular forces due to the addition of a cross-linking agent. The scheme of creating microcapsules in this way is shown in Figure 6 [4,29].

In this method, microcapsules are synthesized in an aqueous solution and at the interface of oil drops. The preparation of alginate microcapsules is accomplished by mixing the encapsulated component (usually hydrophobic drugs) with an organic solvent such as ethanol or acetone to form the inner phase (oil phase) of the microcapsules [103]. The previously prepared mixture is added to the alginate solution containing, among others, Tween 80 as a surfactant. It is then subjected to homogenization or sonication to produce an O/W emulsion. The calcium chloride solution is slowly added to the formed emulsion, which causes the alginate to cross-link and form microcapsules. The core of the so-prepared microcapsules consists of an oily liquid surrounded by a single polymer layer. The microcapsule suspension is equilibrated for several hours. Finally, the solvent is removed under reduced pressure and the microcapsules formed are washed and isolated [42,104,105]. Capsules of different row sizes can be obtained with this method. It has been used for encapsulating, among others, testosterone [106] or essential oils [107].

In the case of the emulsification technique, core/shell microparticles with a liquid core are formed. However, the latest few reports also characterized the AMP with a solid core. For example, in a recent paper by Dubashynskaya et al., microparticles consisting of a core made of partially deacetylated chitin nanowhiskers and an alginate shell were used for the topical drug delivery of metronidazole [108].

## 5. Factors Influencing the Alginate Microparticle Formation Process

During the manufacture of AMP, regardless of the technique used, various factors are key parameters. The alginate concentration, type and concentration of cross-linking vector, type of oil, emulsifiers, pH regulator, agitation speed and cross-linking time influence the quality of alginate microparticles (size, shape, stability and encapsulation efficiency).

### 5.1. Concentration of Sodium Alginate

Chan et al. [109] highlighted the effect of alginate concentration on the size of beads prepared by use of the extrusion method. They observed that the concentration of alginate below 1% resulted in the formation of nonspherical capsules, probably due to the lack of a sufficient number of carboxyl groups for the gelling process. On the other hand, when the concentration of sodium alginate was higher (>5%), the increase in the viscosity of the aqueous phase resulted in larger droplets with broad distribution. This is consistent with the studies by Liu et al. [110], who investigated the effect of alginate concentration on the size of capsules produced with the emulsification technique/internal gelation.

In the case of the microencapsulation of bacteria, Mandal et al. [111], investigated the effect of alginate concentration on the survival of microencapsulated bacteria in the emulsification technique/external gelation. They used alginate concentration in the range of 2–4%. The obtained results show that an increase in alginate concentration results in better resistance to unfavorable conditions in the gastrointestinal tract, which may be due to the more rigid structure of the microspheres with the use of a higher concentration of alginate. Similar results were obtained by Lotfipour et al. [112], who conducted research on the microencapsulation of bacteria with the extrusion technique. They additionally found that the mean bead diameters increased significantly with increasing alginate concentration, which can be attributed to the greater viscosity of the resulting gel. According to research in this area, an increase in the viscosity of the starter gel leads to the production of larger beads with extrusion. Therefore, for a given application, the alginate concentration must be controlled in terms of particle size, shape and particle distribution.

### 5.2. Type and Concentration of Cross-Linking Agent

Alginate gels cross-link in the presence of divalent cations. Chan et al. [109] investigated the effect of various cations on the properties of alginate gels. In the study, they used cations of calcium, barium, strontium, potassium, aluminum, lithium, ammonium and copper. It was found that only a few cations (Ca^2+^, Sr^2+^, Ba^2+^ and Cu^2+^) can form spherical alginate shells. Aluminum ions did not form spherical capsules, while potassium, lithium and ammonium ions did not form gels with alginate under experimental conditions. Earlier studies have shown that several divalent cations can bind to alginates of different affinity in the following order: Mg^2+^ < Mn^2+^ < Zn^2+^, Ni^2+^, Co^2+^ < Fe^2+^ < Ca^2 +^ < Sr^2+^ < Ba^2+^ < Cd^2+^ < Cu^2+^ < Pb^2+^ [113,114]. In addition, Chan et al. [109] investigated the influence of cation type on the strength of formed alginate gel. The gel strength was expressed in Young’s modulus. Cations of copper (802 kPa), in comparison with barium (592 kPa), strontium (464 kPa) and calcium (339 kPa), formed the strongest gels. This is in accordance with results obtained by Harper et al. [113,115]. They concluded that the size of used cation affects the strength of the alginate gel. The larger cations (such as Ba^2+^ and Sr^2+^) provide stronger binding forces in the junction zones, thus forming stronger gels. Although cations of barium and copper formed stronger alginate gels, calcium is the most popular cation used to cross-link alginates. Ca^2+^ is considered clinically safe, readily available and economical [100]. Additionally, the alginate microspheres and microcapsules could be chemically cross-linked by covalent bonds (using, for example, epichlorohydrin) [116,117], offering the potential for microgel preparation with highly controllable porosities and cell interactions [118], or by ionic bonds with cationic polymers such as N,N′-methylene bisacrylamide [119].

Walczak et al. [41] conducted research on the effect of CaCl_2_ concentration on calcium alginate cross-linking using the extrusion method. It has been observed that with an increase in calcium chloride concentration (0.075–0.5 M), the number of microparticles obtained decreased, while their hardness increased. The gel’s hardness increase is a consequence of alginate cross-linking increasing along CaCl_2_ concentration. On the other hand, calcium chloride concentration (1–4%) had no significant effect on the size and an encapsulation efficiency of the alginate beads. Won et al. [120] studied the effect of CaCl_2_ concentration on the efficiency of lipase encapsulation with the extrusion method. They did not observe a significant effect of CaCl_2_ concentration on the immobilization efficiency. These results are similar to those obtained by Lotfipour et al. [112], who studied the influence of calcium chloride concentration on the encapsulation of bacteria in alginate beads with the extrusion technique. It seems that the excess of Ca^2+^ ion concentration above a certain level (3 M) does not affect the formation of gel networks, and as a consequence, the efficiency of encapsulation [120].

### 5.3. Type and Concentration of the Emulsifier

Surfactants pose two functions in emulsification processes. The first one is to lower the interfacial tension between the water and oil phases and to facilitate the dispersion of a viscous solution of alginate in the oil. The second one is to stabilize the emulsion droplets against coalescence [100,110]. Various emulsifiers have been found useful in the emulsifying/gelling processes of alginate emulsions. Table 2 shows examples of emulsifiers used in the production of AMP.

The data in the table show that the most commonly used surfactants in the microemulsion method are nonionic sorbitan esters. This is most likely due to the fact that they are the most popular surfactants used to stabilize microemulsions. Another important parameter in the case of the microparticles obtaining process is the concentration of these emulsifiers.

Liu et al. [110] conducted a study in which they found that the size of calcium alginate capsules in the emulsification technique/internal gelation significantly decreased from 218 µm to 76.7 µm with an increase in the surfactant concentration (Span 80) from 0.5–2.0%. Since the surfactant is adsorbed on the surface of the droplets of the dispersed phase and forms a film to prevent their coalescence, lower surfactant concentration results in not completely covering the surface of the droplets, which declines droplets stability. Coalescence causes larger droplets. On the other hand, emulsifier concentration above 2.0% is of little benefit to the formulation as the oil-soluble acid (glacial acetic acid) should diffuse across the interface of water and oil to initiate the gelling reaction. Therefore, a high concentration of the surfactant may result in resistance to mass transfer to protons, prolonging gelation and resulting in low particle production [110]. A similar finding was presented by Alnaief et al. [122]. An increase in the emulsifier concentration leads to a reduction in the interfacial tensions between the alginate and the oil phase of the emulsion, and thus to the reduction in the average size of the microspheres.

### 5.4. Effect of an Oil Type on Alginate Capsule Properties

Food-grade and mineral oils are commonly applied in the process of alginate microspheres preparation. However, mineral oils have a potentially greater risk to the environment [132], and therefore food-grade oils are used more frequently. Among food-grade oils, the following are used: corn oil [129], canola oil [133], sunflower oil [20], soy bean oil [84] and olive oil [134]. The effect of the oil phase on the properties of microparticles has not been extensively studied. It is reported that the mean particle diameter obtained depends on the viscosity of the oil used; therefore, the choice of oil depends on the required particle size. For example, Wang et al. found that the particles were larger and additionally had a wider particle size distribution when olive oil was used compared with liquid paraffin oil, because the viscosity of olive oil compared with liquid paraffin oil was higher [134].

### 5.5. pH Values

The pH range for encapsulation depends on several parameters such as the type of polysaccharide, the ion vector and the kind of encapsulating agent [100]. In general, the complex of cationic and anionic ions is formed as a result of electrostatic interactions between charged particles [29,135]. The strength of the polyelectrolyte complex is significantly influenced by the pH value of the solution [29,136]. For a given hydrocolloid, the pH range that will be optimal and will provide the greatest number of ionized or protonated groups required for interaction should be checked. In the case of alginate, the pKa ranges from 3.4 to 4.4. When emulsion pH is higher than pKa, alginate carboxylate groups are ionized and electrostatically linked to cationic polymers and calcium ions [29,137]. Additionally, negatively charged carboxylate groups dominate in a weakly acidic environment with a pH value of about 5 [29,138]. Neutral pH values are most appropriate in the case of the encapsulation of living cells. Acid-tolerant cultures, such as lactic acid bacteria, can be immobilized in the lower pH range, down to 5 [139]. In the case of proteins, the isoelectric point must be taken into account [100].

In the case of the emulsification method/internal gelation, an important factor is the selection of the appropriate oil-soluble organic acid. There are many different oil-soluble organic acids that can be used, but acetic, citric and lactic acids are the most common ones [100]. The acid concentration must be carefully calculated to promote the release of calcium from the calcium complex without overdosing, otherwise it may potentially damage the enclosure [139].

### 5.6. Agitation Speed

In addition to the factors related to the composition of the reaction mixture, the technological parameters of the emulsification process also affect the quality of the microcapsules obtained. The literature sources indicate that increasing the mixing speed has a positive effect on reducing the size of microparticles. The high speed of mixing produces finer microparticles, which is related to greater energy that ensures the dispersion between the oil phase and the water phase in an emulsion [140]. The results of Ahmed et al. [141,142] indicate that the rotor speed was the most important parameter for controlling the size of the microparticles. It has been shown that increasing the agitation speed from 200 to 600 rpm generally reduces the size of the microspheres as they produce smaller emulsion droplets due to higher shear forces and increased turbulence in the emulsion. Similar conclusions have been presented by Rodrigues et al. [143], who prepared microspheres containing *Aeromonas hydrophila* bacteria using the emulsification technique/external gelation. As a result of increasing the rotation speed from 500 rpm to 2000 rpm, the average particle size decreased from 230 to 30.1 µm. Moreover, Alnaief et al. [122] made similar conclusions regarding the size of the microparticles. They explained that increasing the agitation speed from 200 to 1400 rpm gave a large energy input in order to create a larger interface area; thus, the dispersed droplets became finer and the microspheres were smaller in size. As reported by Shukla et al., who prepared alginate microspheres containing diloxanide furoate, using the emulsification technique/external gelation increased agitation speed (750–1500 rpm) and had a positive effect on the reduction in microspheres (from 455.92 to 348.24 µm). However, when the mixing speed (1500 rpm) was too high, the formation of irregularly shaped microspheres occurred as an effect of particle aggregation [127,144,145]. Therefore, in order to obtain satisfactory results, it is necessary to select the value of the mixing rate individually, proper to each application.

### 5.7. Cross-Linking Time

Mali et al. drew attention to the effect of different cross-linking times on alginate microspheres loaded with domperidone prepared with the emulsification technique/external gelation. Their results show that increasing the gelation time from 5–15 min slightly increased the size (57.63–58.86 µm) of the alginate microspheres. However, it was found that with increasing cross-linking time, the encapsulation efficiency decreased (from 50.73% to 18.24%). This can be attributed to incomplete emulsification as a result of the higher viscosity of the internal aqueous phase [124]. Gedam et al. reached similar conclusions by preparing alginate microspheres filled with risedronate sodium using the emulsification technique/external gelation. As a result of increasing the cross-linking time from 5 to 10 min, the encapsulation efficiency decreased, and the size of the microparticles increased. Extended cross-linking time may be responsible for relatively greater cross-linking of sodium alginate guluronic acid units, which in turn could increase the viscosity of the preparation and cause the formation of larger microspheres [123,146]. On the other hand, too short cross-linking time may cause incomplete cross-linking of the alginate and, consequently, reduce the efficiency of encapsulation [29].

In turn, Lin et al. prepared alginate beads filled with astaxanthin using the extrusion technique. It was shown that with the increasing cross-linking time (from 15 to 60 min), no significant difference was observed, both in the mean bead size and the microencapsulation efficiency [127]. Similar conclusions were presented by Lotfipour et al. regarding the influence of mixing time on microencapsulation of bacteria with extrusion. Therefore, a short mixing time may not be sufficient to fully cross-link and generate intense electrolyte interactions. Consequently, larger particles with exposed pores are obtained, resulting in compound losses in the microparticle and reduced encapsulation efficiency [29,147,148]. On the other hand, if the mixing time is too long, it may increase the viscosity of the alginate phase, reduce the voids in the alginate matrix and increase the porosity and thus the leakage of active substance molecules from the alginate droplets to the medium [29,149,150].

## 6. Application of Alginate Microparticles

The AMP are very popular vehicles used for the encapsulation of active substances in food and pharmaceutic products (Table 3 and Table 4). Primarily, the carriers were applied in the food industry and later on in pharmaceutical applications. In the case of cosmetics, alginate microparticles are not very popular yet, but as naturally originating, nontoxic, biodegradable and biocompatible systems, additionally showing a moisturizing and protective effect on skin, they have potential to grow their application [151].

### 6.1. The Application of AMP as Active Substance Carriers in the Food Industry

Alginates are very popular polymers in food industry. They are applied as thickening, gel-forming and stabilizing agents. Moreover, the alginate-based formulations, being biocompatible, biodegradable and nontoxic, are used as active compounds carriers.

With regard to the actives encapsulated in AMP, the probiotic micro-organisms are the most often entrapped [152,153,154,155,156,157,158]. The data collected in Table 3 show that the most common bacteria enclosed in alginate carriers are probiotics of the genus *Lactobacillus*, among others such as *L. plantarum*, *L. acidophilus* and *L. reuteri*. In the alginate systems, the probiotics have been incorporated into various formulations such as freeze-dried powders, tablets, pellets and microcapsules [159,160]. The microencapsulation process not only protects probiotics against unfavorable conditions in the digestive tract but also improves their viability and increases survival rate [30,152,161]. It should be underlined that the size of probiotic bacteria usually ranges from 1 to 4 μm [162]. For such reason, it would not be possible to encapsulate them in nano-sized carriers.

**Table 3:** The application of alginate carriers in the food industry.

Capsules Components	Concentration		Active Ingredients	Encapsulation Technique	References
	[%]	[M]			
sodium alginate chitosan calcium chloride	0.05 0.05 -	- - 0.0002	Nisin	Extrusion	[20]
sodium alginate guar gum calcium chloride	1.5–2.5 0.2–0.6 -	- - 0.1	Nisin	Extrusion	[163]
sodium alginate polyvinyl alcohol whey protein concentrate calcium chloride	31.2 31.2 18.8 5	- - - -	Sea buckthorn berries bio-oil and amaranth seeds bio-oil	Extrusion	[164]
sodium alginate calcium chloride fructooligosaccharides, isomaltooligosaccharides or peptide coated	1–3 - -	- 0.1 -	*L. acidophilus*, *L. casei*, *B. bifidum* and *B. longum*	Extrusion	[165]
sodium alginate inulin or Jerusalem artichoke calcium chloride chitosan coated	3 3 - -	- - 0.1 -	*L. acidophilus*	Extrusion	[152]
sodium alginate starch lecithin calcium chloride	2 2 0–4 -	- - - 0.1	*L. casei*, *L. plantarum*, *L. acidophilus*, *L. gasperi*, *L. bulgaricus*, *B. adolescenti * and *L. lactis*	Extrusion	[166]
sodium alginate xanthan gum fructose maltose glycerol calcium chloride	2 0.15 3 3 5.5	- - - - -	*L. plantarum*	Extrusion	[167]
sodium alginate sugarbeet calcium chloride	2 2 -	- - 0.1	*Staphylococcus* *succinus* and *Enterococcus fecium*	Extrusion	[168]
sodium alginate calcium chloride	2 0.5	- -	*Saccharomyces cerevisiae*	Extrusion	[169]
sodium alginate guar gum calcium chloride whey protein and chitosan coated	3 5 - 2	- - 0.1 -	*Yarrowialipolytica*, *Kluyveromyces lactis*, *Lipomycesstarkeyi*, *Saccharomycopsisfibuligera* and *Brettanomycescustersianus*	Extrusion	[170]
sodium alginate calcium chloride	1.875 -	- 1.5	*L. bulgaricus * and *Streptococcus thermophilus*	Extrusion	[171]
sodium alginate calcium chloride	1.875 -	- 1.5	*Streptococcus lactis*, *Streptococcus lactis* subsp. *diacetylactis* and *Streptococcus cremoris*	Extrusion	[172]
sodium alginate calcium chloride	3 -	- 0.5	*L. reuteri* and *B. longum*	Extrusion	[173]
sodium alginate calcium chloride	1 -	- 0.1	*Streptococcus lactis* and *Streptococcus cremoris*	Extrusion	[174]
sodium alginate calcium chloride	0.75–2 -	- 0.1; 0.2; 1	*L. acidophilus*	Extrusion	[175]
sodium alginate locust bean gum xanthan gum calcium chloride chitosan coated	2 - - - -	- - - 0.1 -	*L. rhamnosus*	Extrusion	[176]
sodium alginate calcium chloride	3 -	- 0.15	*L. plantarum*	Extrusion	[177]
sodium alginate pancreatic digested casein fructooligosacharides isomaltooligosaccharides calcium chloride	1–3 0–1 0–3 0–3 -	- - - - 1	*L. casei*, *L. acidophilus*, *B. longum* and *B. bifidum*	Extrusion	[178]
sodium alginate calcium chloride	3 -	- 0.5	*L. reuteri*	Extrusion	[179]
sodium alginate calcium chloride	2 0.5; 0.8	- -	*L. plantarum*	Extrusion	[180]
sodium alginate Hi-maize resistant starch calcium chloride	2 2 -	- - 0.1	*L. acidophilus*	Extrusion	[181]
sodium alginate fruktooligosacharides pancreatic digested casein calcium chloride	1–3 0–3 0–1 -	- - - 0.1	*B. bifidum*	Extrusion	[182]
sodium alginate calcium chloride	3 -	- 0.5	*L. reuteri*	Extrusion	[183]
sodium alginate starch calcium chloride	2 2 -	- - 0.5	*L. reuteri*	Extrusion	[183]
sodium alginate glycerol xanthan gum Tween 20 calcium chloride chitosan coated	2 5 0.26 0.1 - 0.8	- - - - 0.5 -	*L. bulgaricus*	Extrusion	[128]
sodium alginate calcium chloride	2 -	- 1	*L. acidophilus* and *B. bifidum*	Extrusion	[184]
sodium alginate or palmitoylated alginate calcium chloride	2 30	- -	*B. longum*	Extrusion	[185]
sodium alginate calcium chloride	1 -	- 0.1	*L. lactis* subsp. *cremoris*	Extrusion	[186]
sodium alginate glycerol xanthan gum calcium chloride chitosan or gelatin coated	1–3 5 0.9 - -	- - - 1 -	*L. plantarum*	Extrusion	[187]
sodium alginate calcium chloride	- 2	0.1 -	*L. acidophilus*	Extrusion	[188]
whey proteins concentrate sodium alginate calcium chloride	2.5–4 0.125 -	- - 0.1	*L. acidophilus*	Extrusion	[188]
sodium alginate whey protein isolate	- -	- -	*L. plantarum*	Spray-drying	[189]
sodium alginate calcium chloride	2 -	- 0.1	*L. rhamnosus*	Spray-drying	[190]
sodium alginate chitosan Tween 40 calcium chloride	1–2 - 0.5–1.5 -	- - - 0.5	Coriander essential oil	Emulsification (external gelation)	[97]
sodium alginate and alginate-resistant starch sunflower oil Span 80 Tween 80 calcium chloride	1 - 1 1 25	- - - - -	Nisin	Emulsification (external gelation)	[19]
sodium alginate CaCO_3_ Span 80 polyglycerol polyricinoleate (PGPR) sunflower oil glacial acetic acid Tween 20 calcium chloride	2 - - 4–15 - - - 0.03–01	- - - - - - - -	Cocoa extract	Emulsification (internal gelation)	[131]
sodium alginate vegetable oil Tween 80 calcium chloride	- - - -	- - - 0.0625	*L. acidophilus* and *L. rhamnosus*	Emulsification (external gelation)	[191]
sodium alginate Hi-maize resistant starch Tween 80 calcium chloride	2 2 0.02 -	- - - 0.1	*L. acidophilus* and *Bifidobacterium* spp.	Emulsification (external gelation)	[83]
sodium alginate guar gum xanthan gum locust bean gum carrageenan gum vegetable oil Tween 80 calcium chloride	3 - - - - - - -	- - - - - - - 0.01	*L. rhamnosus*, *B. longum*, *L. salivarius*, *L. plantarum*, *B. lactis*, *L. paracasei*, *L. acidophilus * and *B. bifidum*	Emulsification (external gelation)	[192]
sodium alginate Hi-maize resistant starch corn oil Tween 80 calcium chloride chitosan coated	3 2 - - - -	- - - - 0.1 -	*L. acidophilus* and *L. casei*	Emulsification (external gelation)	[129]
sodium alginate faxseed mucilage canola oil Tween 80 calcium chloride	1–3 0.9 - 0.05 -	- - - - 0.15	*L. casei*	Emulsification (external gelation)	[133]
sodium alginate vegetable oil Tween 80 calcium chloride	3.6 - 2 -	- - - 0.05	*L. bulgaricus*	Emulsification (external gelation)	[193]
sodium alginate Hi-maize resistant starch canola oil lecithin calcium chloride	2 2 - 0.02 -	- - - - 0.1	*L. casei * and *B. lactis*	Emulsification (external gelation)	[194]
sodium alginate corn oil Tween 80 calcium chloride	3 - 0.02 -	- - - 0.1	*L. reuteri*	Emulsification (external gelation)	[174]
sodium alginate Hi-maize starch calcium chloride	2 2 -	- - 0.1	*L. acidophilus * and *B. lactis*	Emulsification (external gelation)	[195]
sodium alginate soybean oil Tween 80 calcium chloride	2–4 - 0.2 -	- - - 0.05	*L. casei*	Emulsification (external gelation)	[111]
sodium alginate sodium citrate calcium citrate canola oil glacial acetic acid	2 0.1 1 - 0.667	- - - - -	*Lactococcus lactis* subsp. *cremoris*	Emulsification (internal gelation)	[196]
3% sodium alginate corn oil Tween 80 calcium chloride	3 - 0.02 -	- - - 0.1	*L. reuteri*	Emulsification (external gelation)	[183]
sodium alginate starch corn oil Tween 80 calcium chloride	2 2 - 0.02 -	- - - - 0.1	*L. reuteri*	Emulsification (external gelation)	[183]
sodium alginate corn starch soybean oil Tween 80 calcium chloride	4 2 - - -	- - - - 0.1	*L. acidophilus*	Emulsification (external gelation)	[197]
sodium alginate CaCO_3_ canola oil Tween 80 glacial acetic acid	1.5 - - 1.5 -	- - - - -	*B. animalis* subsp. *lactis*	Emulsification (internal gelation)	[198]

### 6.2. The Application of AMP as Active Substance Carriers in the Pharmaceutical Industry

Alginate-based formulations as delivery systems have been originally developed for pharmaceutical applications to provide controlled release and improve the stability of drugs. Moreover, as in the case of food applications, the probiotic micro-organisms are mostly entrapped in such carriers (Table 4).

**Table 4:** The application of alginate carriers in the pharmaceutical industry.

Capsules Components	Concentration		Active Ingredients	Encapsulation Technique	References
	[%]	[M]			
sodium alginate citric acid dicalcium phosphate succinic acid latex bovine serum albumin	2–4 0.6 0.2 4 0.05–0.5 0.15	- - - - - -	Cellulase	Spray-drying	[70]
sodium alginate pectin Tween 80	3 3 -	- - -	Carvacrol	Spray-drying	[71]
sodium alginate	2	-	Insulin	Spray-drying	[72]
sodium alginate Tween 80 succinic acid calcium phosphate dibasic dihydrate	4 - 2 0.2	- - - -	Corn oil	Spray-drying	[69]
sodium alginate calcium chloride chitosan coated	2 2.5 -	- - -	Bovine serum albumin	Spray-drying	[25]
sodium alginate sun flower oil oleic acid ester calcium chloride	3 - 1 4	- - - -	Catechin	Extrusion	[18]
sodium alginate pectin glycerol Tween 80 calcium chloride	0.5–2 2 1 0.1 5	- - - - -	α-tocopherol	Extrusion	[199]
sodium alginate calcium chloride	1 2.5	- -	Invertase	Extrusion	[200]
sodium alginate calcium chloride whey proteins coated	20 - 20	- 0.1 -	*L. plantarum*	Extrusion	[201]
sodium alginate alginate/psyllium blend alginate/fenugreek blend calcium chloride	2 1.5/0.5 1.5/0.5 4	- - - -	*L. plantarum*	Extrusion	[202]
xanthan gum sodium alginate Tween 80 calcium chloride chitosan coated	0.15 1.8 1 - -	- - - 0.1 -	*L. plantarum*	Extrusion	[203]
sodium alginate calcium chloride chitosan coated	2 - -	- 0.1 -	*L. acidophilus*, *B. bifidum* and *L. casei*	Extrusion	[204]
sodium alginate calcium chloride	2–4 -	- 0.1	*B. longum*	Extrusion	[205]
sodium alginate calcium chloride	1.8 -	- 0.1	*Saccharomyces boulardii*	Extrusion	[206]
sodium alginate calcium chloride poly-L-lysine and alginate coated	1.5 - 0.1 and 0.1	- 0.1 -	*L. reuteri*	Extrusion	[207]
sodium alginate oligosaccharides (GOS, FOS, IMO, XLO) calcium chloride	2 1.8 2	- - -	*L. fermentum*	Extrusion	[208]
sodium alginate zein calcium chloride	1.4 1–9 -	- - 0.1	*B. bifidum*	Extrusion	[209]
sodium alginate Tween 80 glycerol calcium chloride	2 - - -	- - - 2	Testosterone	Emulsification (complexation)	[106]
sodium alginate chitosan Tween 80 ethanol calcium chloride	0.3–0.6 0.3–0.6 1 - 0.67	- - - - -	Lemongrass oil and turmeric oil	Emulsification (complexation)	[107]
sodium alginate CaCO_3_ soybean oil glacial acetic acid Tween 80 chitosan coated	1.5 - - - 1 0.4	- 0.000625 - - - -	*B. longum*	Emulsification (internal gelation)	[84]
sodium alginate corn starch vegetable oil Tween 80 calcium chloride	1 2 - 0.2 -	- - - - 0.1	*L. casei* and *B. bifidum*	Emulsification (external gelation)	[210]
gelatin genipin tempered oil Span 85 calcium chloride sodium alginate coated	13 - - 0.5 - 1	- 0.00125 - - 0.05 -	*B. adolescentis*	Emulsification (external gelation)	[211]

### 6.3. The Application of AMP as Active Substance Carriers in the Cosmetic Industry

In the case of cosmetics, alginate microparticles are not very popular yet, but as naturally originating systems additionally showing a moisturizing and protective effect on skin, their application is still growing. Currently, in the case of skin care products, AMP are used as carriers, both to protect unstable active compounds and to increase products’ efficacy [212,213,214,215]. Additionally, AMP are used to enclosed unpleasant-smelling substances and, as a result, to obtain products with little odor or completely without it [214]. The examples of AMP cosmetic applications are presented in Table 5.

The data presented in Table 3, Table 4 and Table 5 show that extrusion, among others, is the most commonly used method for encapsulating substances in alginate systems, while the least used technique is spray drying. It is probably, due to the fact, that extrusion is a cheap method in which there is no need to use additional components, such organic solvents, emulsifiers, or large amounts of oil.

Comparing the methods of probiotics microencapsulation (Table 3, Table 4 and Table 5), it is clear that extrusion is the most popular technique, probably because it does not damage bacteria cells and gives high probiotic viability. In turn, external gelation is the most frequently chosen method of emulsification process. This may be due to the fact that during external gelation, it is not necessary to use any additional acid, which could also cause interactions with microorganisms and, consequently, their reduced survival in microparticles.

### 6.4. Other AMP Applications

In recent years, the use of alginate microcapsules has been intensively developed in agriculture [219,220,221] for encapsulating plant-growth-promoting bacteria. Plants can grow better in the presence of plant probiotic bacteria because these bacteria carry out a number of various important functions, such as nitrogen fixation and mineral dissolution, among others. Other designs include the application of AMP as phase-change materials for thermal energy storage [222]. The urgent need to meet increasing energy demand and mitigate the environmental impact of fossil fuel combustion is driving large-scale research into efficient and sustainable methods of storing thermal energy. Among various technologies, systems using phase-change materials present one of the most promising strategies. Another potential application of alginate microparticles is metallic protection. The common application includes self-healing coatings for corrosion prevention in metal substrates. Hia et al. developed surface-modified alginate multicore microcapsules for metallic protection [223]. AMP are also used in the separation of metal ions, such as Mo(VI) [224], Sb(III) [225], Pu(IV) [226], Platinum Group Metals [227] and Cu^2+^ [228], among others, and for photocatalytic wastewater treatment [229].

### 6.5. Advantages and Disadvantages of Alginate Microparticles

The alginates used as raw material to obtain the microparticles show a lot of advantages but also a few disadvantages. Among the advantages of using AMP as a carrier of active compounds, it should be mentioned that:

- Alginates form a highly versatile, biocompatible, biodegradable and nontoxic matrix, effectively protecting active components against external factors such as heat and moisture, thereby enhancing stability and bioavailability [131,152,200];
- Alginates show a prebiotic effect of low molecular weight alginates and their ability to extend the product shelf life [131,230] of beverages [231], yoghurt [232], sausages [233,234], tuna burgers [235], cheese [236], apple juice [237] and ice-cream [238], among others;
- There are mild microencapsulation conditions and no high temperatures (emulsification and extrusion), which is especially important in the case of the encapsulation of bacteria [200], because they are very sensitive to high temperatures;
- Alginates form highly stable particles in the spray-drying technique [70], however, the use of high temperatures in this method when encapsulating probiotics may reduce their effectiveness and number [153];
- Alginates improve the stability and increase the efficiency of delivery to the body of microencapsulated active compounds [72], including probiotic microorganisms;
- Alginates protect bacterial cells against the external environment [152], reduce susceptibility to contamination and protect against damage [205], such as the presence of gastric juices, acidity and low pH [239,240], and in the case of cosmetics, protect probiotics against the effects of preservatives;
- Alginates increase the survival of probiotic bacteria both in the product and in the gastrointestinal tract [165,168,191], which allows them to reach the intestines, and thus has a positive effect on the intestinal microbiome [241,242].

On the other hands, among the disadvantages of AMP, attention should be paid to:

- The low efficiency of capsules loaded with bacteria, difficulties in application on an industrial scale due to the high cost of bacteria and the microencapsulation techniques [72];
- Susceptibility to the acidic environment of AMP loaded with bacteria, which reduces the efficiency of encapsulation, such as a result of gastric juices [133,201,243];
- The low mechanical strength of AMP and large pore size, which may cause the leakage of biomolecules from microcapsules [200,244,245];

These disadvantages can be overcome, and the encapsulation efficiency can be increased by using a coating material, e.g., chitosan [152], a peptide or fructooligosaccharides [165].

## 7. Summary

The manuscript presents a review of the current state of alginate polymer applications in the form of alginate microparticles (AMP) in the food, pharmaceutic and cosmetic industries. The most important properties of alginates, including safety and biocompatibility, were described. The AMP preparation methods, their characteristics and industrial applications were discussed. This overview provides an understanding of encapsulation techniques (such as extrusion, emulsification and spray-drying processes) and the process parameters that are critical to microcapsules production and affect particle size, morphology and encapsulation efficiency. The influence of the microencapsulation process parameters on microparticle quality was discussed. Finally, a summary of the active compounds encapsulated in the alginate carriers was presented, and the advantages and some disadvantages of the alginate particles as delivery systems were summarized.

It was shown that alginate as natural biopolymer forms a highly versatile, biocompatible, biodegradable and nontoxic matrix, effectively protecting the active components against external factors, and thereby enhancing their stability and bioavailability. However, there are only few studies devoted to the use of alginate capsules as carriers of active substances in cosmetic products, despite the fact that alginate carriers can be an ideal solution, especially as probiotic carriers, in the case of skin-microbiome-friendly cosmetics.

In addition, given the various applications in which AMP are already used for due to their attractive physicochemical properties and the continuously evolving methods of producing them, alginates will make a significant contribution to revolutionizing research in the future.

Another future interest is the development of the alginate microencapsulation techniques. The upcoming process for the production of microcapsules and microspheres, with great potential also in alginate microcapsule production, is the microfluidic technique. The process allows the preparation of microgels with a wide range of morphologies and allows the continuous fine-tuning of the shape of the microparticles by simply changing the gelation conditions, e.g., the viscosity of the gelation bath, collecting height or interfacial tension [246,247].

## Figures and Tables

**Table 1:** Advantages and disadvantages of alginate microencapsulation methods [3,79,84,88,89,90].

Method	Advantages	Disadvantages
Extrusion	- simple and cheap method - no use of harmful solvents - no use of large amounts of oil and emulsifier - in the case of probiotics, no damage to the probiotic cell and high viability	- slow formation of microparticles → a problem in a large-scale application - limited choice of encapsulated actives
Emulsification	- the possibility of encapsulating both hydrophilic and hydrophobic ingredients - high process efficiency - the possibility of obtaining smaller microparticles than in extrusion and spray drying methods - easy to scale up and fewer restrictions on preparation devices - high survival rate of probiotics	- higher costs than in the case of extrusion due to the use of a large amount of oil and emulsifier - toxicity of organic solvents and droplet agglomeration due to solvent removal in the case of microcapsules formed from a complex at the interface of emulsion droplets
Spray-drying	- low cost - high efficiency and quality of the obtained microparticles - adaptable to most typical industrial devices	- nonuniform capsules - material losses due to the adherence of substance particles to the drying chamber - limitations in the choice of coating material - in the case of probiotics, the loss of viability due to the high-temperature drying requirement

**Table 2:** Surfactants used in the production of alginate microparticles.

Trade Name	Concentration of Emulsifier [%]	Encapsulation Techniques	References
Lecithin	0.1; 0.5; 1.0; 2.0	Emulsification/internal gelation	[121]
Span 80	0.5–2.0	Emulsification/internal gelation	[110]
Span 80	0–3.0	Emulsification/internal gelation	[122]
Span 80	2.0	Emulsification/external gelation	[123]
Span 80	2.0	Emulsification/external gelation	[124]
Span 85	2.0	Emulsification/external gelation	[125]
Span 85	-	Emulsification/internal gelation	[126]
Tween 20	0.5; 2.0	Extrusion	[127]
Tween 20	0.1	Extrusion	[128]
Tween 80	0.02	Emulsification/external gelation	[83]
Tween 80	-	Spray-drying	[69]
Tween 80	-	Spray-drying	[71]
Tween 80	-	Emulsification/complexation	[106]
Tween 80	0.2	Emulsification/external gelation	[129]
Mixture: Span 80 and Tween 80	1.0 Span 80 and 1.0 Tween 80	Emulsification/external gelation	[19]
Mixture: Span 80 and Tween 80	1.0 (mixture 60/40)	Emulsification/internal gelation	[130]
polyglycerol polyricinoleate (PGPR)	4.0–15.0	Emulsification/internal gelation	[131]

**Table 5:** The application of alginate carriers in the cosmetic industry.

Capsules Components	Concentration		Actie Ingredients	Encapsulation Technique	References
	[%]	[M]			
sodium alginate Tween 20 calcium chloride	1–3 0.5; 2 2; 10	- - -	Astaxanthin	Extrusion	[127]
sodium alginate calcium chloride	- -	- -	Lactic acid bacteria (*Lactobacillus)*	Extrusion	[216]
sodium alginate solution ethanol Tween 80 calcium chloride	- - - -	- - - -	Tumeric oil	Emulsification	[217]
sodium alginate pectin Tween 80 calcium chloride	0.5–2.5 5 0.1 5	- - - -	Vitamin E	Emulsification	[218]

## Data Availability

Not applicable.
